# CFD simulation study on wind load of perforated traffic sign board

**DOI:** 10.1371/journal.pone.0240927

**Published:** 2020-10-22

**Authors:** Jinqiang Xu, Hai Xu, Chenghong Zeng, Chengguang Xie, Jiangang Guo

**Affiliations:** 1 College of Transportation Engineering, Chang’an University, Xi’an, Shaanxi, China; 2 College of Transportation and Civil Engineering, Fujian Agriculture and Forestry University, Fuzhou, Fujian, China; Tongii University, CHINA

## Abstract

Traffic sign boards are often blown away by strong winds, seriously endangering the safety of vehicles and pedestrians. To increase their resistance to strong winds, sign boards are perforated. Using computational fluid dynamics simulations, the wind load resistance of traffic signs with holes was optimised. By comparing the solutions to different turbulence models with empirical results, it was found that the simulation results of the re-normalisation group (RNG) model have the smallest error. Therefore, the RNG model is used to simulate the wind load of traffic sign boards with different perforation diameters and different hole spacings under different wind speeds. By analysing the wind pressure distribution on the surface of the perforated traffic sign board, the perforation scheme for different regions of the sign board under different wind loads was obtained. The results show that reasonable perforation diameters and hole spacings can reduce the wind load and improve the wind load resistance of sign boards. This study provides decision-makers with useful information for installing traffic signs in areas affected with strong winds, thereby improving the wind resistance of traffic signs and ensuring traffic safety.

## 1. Introduction

As an important part of road infrastructure, traffic signs convey essential information in terms of guidance, restrictions, warnings, or instructions to drivers and pedestrians [1]. In some coastal areas and flat areas, large-scale guide signs are easily damaged by strong winds, which seriously affects road traffic safety. In real-world engineering applications, designers improve the wind load resistance of the sign boards by making holes and slots on these boards [2]. However, the implementation of these measures generally lacks a theoretical basis and relies on the designer's subjective feelings, which often leads to problems, such as structural instability or improper visibility of the sign board and cannot achieve the expected anti-wind load effect. Therefore, the wind-load countermeasures applied to sign boards need a scientific and feasible plan.

Aiming at the problem of insufficient wind load resistance of traffic sign boards, some experts and scholars analysed the force of the support structure under wind load by optimising the support structure of the sign board [3–8]. In addition, they considered the effect of large vehicles passing by. With regards to the influence of the aerodynamic effect on sign boards [9], a new sign board design scheme was proposed [10] that separates the sign board and the supporting structure. However, the optimal design of the sign board structure is limited by the support steel, and the wind load resistance of the sign board is proportional to the cost of the steel. Therefore, improving the wind load resistance of the sign board as much as possible on the original basis is particularly important.

Other experts and scholars have considered studying the characteristics of wind load on the sign board surface. Existing studies have rarely used traffic sign boards as the research object, instead of focusing mainly on objects such as billboards and other buildings. The theoretical basis of the research is fluid mechanics, and the methods of collecting experimental data are wind tunnel experiments and computational fluid dynamics (CFD) simulation experiments. The research on fluid mechanics is mainly divided into two parts. Firstly, the theory of fluid mechanics is used to establish a mathematical model of turbulence and explain the operation mechanism of the wind field. Ismail et al. [11] established a calculation model for the turbulent flow stage with wind tunnel experiments. Similarly, Letchford et al. [12] conducted theoretical analysis and mathematical modelling of wind loads on the sign boards to simulate the wind load in the natural environment. To analyse the action mechanism, Liu et al. [13] used mathematical modelling methods to improve the model of wind tunnel experiments. They also set up a wind tunnel laboratory where the actual structures reduced by a certain proportion were replicated and placed in the wind tunnel to conduct wind pressure testing and obtain experimental data to verify the accuracy of the turbulence model.

Secondly, wind tunnel experiments and CFD simulation experiments are generally used to evaluate wind loads on buildings, and to determine wind pressure, acceleration, and other data [14–16]. Chen et al. [17, 18] studied the effect of complex terrain and low-level wind effects of buildings on the wind characteristics of the glide paths at the North Runway of the Hong Kong International Airport and analysed the effects of terrain and buildings on the wind characteristics, including turbulence intensity, turbulence integral scale, mean wind speed, mean crosswind speed, and mean headwind speed. Winkelmann et al. [19] used wind tunnel experiments to analyse the structure of buildings under wind loads. Huang et al. [20] studied the influence of plate spin geometry, Reynolds number, and rotation direction through wind tunnel experiments. Finally, with the development of computers, computational fluid dynamics (CFD) was extended to these experiments. CFD simulation software is used to simulate the wind environment and verify the accuracy of the simulation experiment through wind tunnel experimental data [11, 21–26]. The development of CFD has made it more convenient to study wind loads on sign boards. With CFD simulation software, the sign board is simulated and evaluated in the wind field environment [27, 28], the aerodynamic effects and wind pressure distribution characteristics of the sign board surface are studied [29–32], and wind-induced response analysis is carried out [33, 34]. Besides, Chao et al. [35] demonstrated the feasibility of CFD simulation through the theoretical calculation method of wind load on traffic sign boards. Shirzadi et al. [36] analysed steady-state the Reynolds average NS model (RANS), Large eddy simulation (LES), and wind tunnels using CFD. The difference between the simulation and experimental results provides a theoretical basis for the subsequent CFD simulation of the sign board.

Pure theoretical fluid mechanics, wind tunnel experiments, and CFD are the three methods of wind load research. CFD is a branch of mechanics that numerically analyses physical phenomena such as fluid flow and heat conduction using computer numerical simulation and visual processing. There are two traditional methods for studying fluid problems, namely pure theoretical fluid mechanics and experimental fluid mechanics. The results of pure theoretical fluid mechanics are generally universal, which provides a theoretical basis for experimental design and new CFD algorithms. The measurement results of experimental fluid mechanics are more authentic and credible and form the basis for studying fluid mechanics problems. With advances in computer technology, CFD has overcome some shortcomings of pure theoretical fluid mechanics and experimental fluid mechanics. For example, the CFD method is low cost and less time-consuming and can accurately predict the flow process and easily obtain data in the flow field. However, it cannot be arbitrarily assumed that the future development of CFD will replace experimental and theoretical analysis. The three analytical methods of fluid mechanics have their advantages and disadvantages, and they are all means for studying flow problems. The relationship between the three is complementary. Therefore, in this study, the empirical formula for wind load on a traffic sign board was adopted to verify the CFD simulation results and adjust the simulation parameters so that the model achieves the desired effect.

In this paper, the CFD simulation of traffic sign boards is carried out using ANSYS Fluent. The results of different turbulence models were compared with the empirical formula for traffic sign wind load [37]. The turbulence model with the minimum error of simulation results was selected for fluid analysis. Then traffic sign models with different perforation diameters and spacings were established to simulate the wind load of the optimal turbulence model. The wind pressure data on the sign surface was extracted by CFD-Post for studying the characteristics of wind pressure changes. Through Fluent fluid analysis software, the turbulence model with the minimum error was adopted to carry out wind load simulations for traffic sign boards with different perforation diameters. The wind pressure data on the surface of the sign board was extracted and then the effect of different perforation diameters on the wind pressure distribution on the surface of the sign board was studied. The relationships between the diameter of the perforation, the wind speed, and the drop in the wind load of the sign board with perforations were explored and the best perforation diameters for different areas on the surface of the sign board were determined. A round hole with a diameter of 90 mm was selected for simulating the wind load of the sign board under different perforation spacings. The variation in the wind load drop of sign boards with different hole spacings is obtained through simulation experiments. The best hole spacing range, for different areas of the sign board were determined. The optimised perforated traffic sign can not only improve the wind load resistance of the sign board but also reduce the strength requirements of the supporting parts to some extent, making it easier to design and install the structure.

## 2. Theory and experiment

### 2.1. Theoretical method

The purpose of CFD simulation is to determine the approximate solution of the fluid control equation by using the fast computing power of the computer. Computational mathematics is used to discretise the flow control equations to obtain the flow of physical quantities at discrete points in space and time. The governing equations of fluid motion include the mass conservation equation, the momentum conservation equation, and the energy conservation equation. This experiment uses the differential expression of the momentum conservation equation, namely, the Reynolds average NS model (RANS) method, which is one of the fundamental equations solved by Fluent. In Fluent software, the k-ε model, k-ω model, and the Reynolds stress model are all RANS models. The commonly used applications of the RANS turbulence model are shown in Table 1.

**Table 1:** Applications of RANS turbulence model.

turbulence model	Suitable conditions
Spalart-Allmaras	The calculation amount is small, and the effect is better for some complicated boundary layer problems. The calculation results have not been extensively tested and lack sub-models.
Standard k-ε	Many applications, moderate computation, more data accumulation, and considerable accuracy. The simulation results of complex flows with large curvature, strong pressure gradient, and rotation problems are insufficient.
RNG k-ε	It can simulate the jet impingement, separation flow, secondary flow, cyclone, and other medium complex flows. It is restricted by the assumption that the eddy viscosity is isotropic.
Realizable k-ε	Basically the same as RNG k-ε, it can also better simulate the circular hole jet problem. It is restricted by the assumption that the eddy viscosity is isotropic
Standard k-ω	For the wall boundary layer, free shear flow, low Reynolds coefficient performance is better. It is suitable for boundary layer flow and separation and rotation in the presence of a backpressure gradient.
SST k-ω	Basically the same as the standard k-ω. It is not suitable for free shear flow because of the muscular wall distance dependence.
Reynolds Stress	It is suitable for curved pipe, rotation, swirl combustion, cyclone separation, and other flows. It takes more CPU time and memory and is difficult to converge

The wind is produced by airflow, and its flow state is classified into laminar flow, excessive flow, and turbulent flow. When the flow rate is very small, the fluid flows in layers and does not mix, called laminar flow. As the flow rate increases, the streamlines of the fluid become wavy and begin to wobble, which is called excessive flow. As the flow rate continues to increase, the streamlines are no longer visible, laminar flow is destroyed, and adjacent flow layers are mixed, known as turbulence. Turbulent and laminar flow states are usually judged by the Reynolds number.

The Reynolds number is given as

$$Re=\frac{\rho \upsilon L}{\mu }$$ (1)

where *ρ* represents fluid density, *υ* represents fluid velocity, *L* represents the solid characteristic length, and *μ* represents fluid dynamic viscosity.

Within the normal distance near a wall, the fluid velocity decreases from a relatively large value to the same as the wall velocity. Therefore, for the calculation of the area near the wall, the wall function or the encryption mesh method should be used to improve the solution accuracy of the wall layer. This study involves a boundary layer problem for a flat board. As the dimensionless wall distance, *y*^*+*^ in the wall function method is reflected in the calculation of the height of the first grid node in the grid division process. By estimating the *y*^*+*^ value, the height of the nodes in the first layer of the grid can be calculated and compared with the expected requirements. If there is a big difference, the estimated *y*^*+*^ value needs to be calculated again. The grid height *y* of the first layer is calculated as follows:

$$y=\frac{y^{+}\mu }{\upsilon_{\tau }\rho }$$ (2)

where *y*^+^ represents the dimensionless wall distance, *μ* is the hydrodynamic viscosity, *υ_τ_* is the wall shear stress estimation, and *ρ* is the medium density.

The wall shear stress estimation is shown as

$$\upsilon_{\tau }=\sqrt{\frac{\tau_{\omega }}{\rho }}$$ (3)

where *τ_ω_* is the wall shear stress, and *ρ* is the medium density.

When the turbulence model is used in the calculation model, the boundary turbulence intensity needs to be set. Some physical quantities in the turbulence boundary are calculated as follows:

The turbulence intensity is

$$i=0.16{\left(Re\right)}^{-1/8}$$ (4)

where *Re* is the Reynolds coefficient.

The turbulence scale is calculated as

l=0.07L (5)

where *L* is the solid characteristic length.

In the experiment, the empirical formula for the wind load *F* on traffic sign boards for checking CFD simulation results is derived from Manual on Layout of Highway Traffic Signs and Markings [37] and calculated as follows:

$$F=\gamma_{O}\gamma_{Q}(\frac{1}{2}\rho Cv^{2})WH$$ (6)

where *γ_O_* is the structural coefficient of cantilevered traffic signs and portal traffic signs on the expressway, and the value is 1. *γ_Q_* is the variable load, mainly wind load, and is equal to 1.4. *ρ* is the air density. *C* is the wind force coefficient and is equal to 1.2. *v* is the wind speed. *W* is the width of the sign board and *H* is the height of the sign board.

### 2.2. Experimental details

In the experiment, a large layout traffic sign is used and the cantilever guide sign is used for modelling. Bradbury et al. [38] pointed out that when the thickness to height ratio of the board is less than 0.33, the influence of the board thickness can be ignored. Therefore, the size of the sign board model is 6 meters in width and 4 meters in height, with negligible thickness.

Considering that the wind speed of traffic signs designed under wind loads is not less than 22 m/s, the wind speed in the CFD simulation is set to six different speeds: 22 m/s, 25 m/s, 30 m/s, 35 m/s, 40 m/s, and 45 m/s. At 20 degrees Celsius, the lowest wind speed is 22 m/s, the air density is 1.208 kg/m^3^, the aerodynamic viscosity is 1.809•10^−5^, and the Reynolds number is 8.815•10^6^. For external flows, turbulence is defined as having a Reynolds coefficient greater than 500000, so the turbulence model should be selected for the CFD wind load simulation.

**Fig 1:**
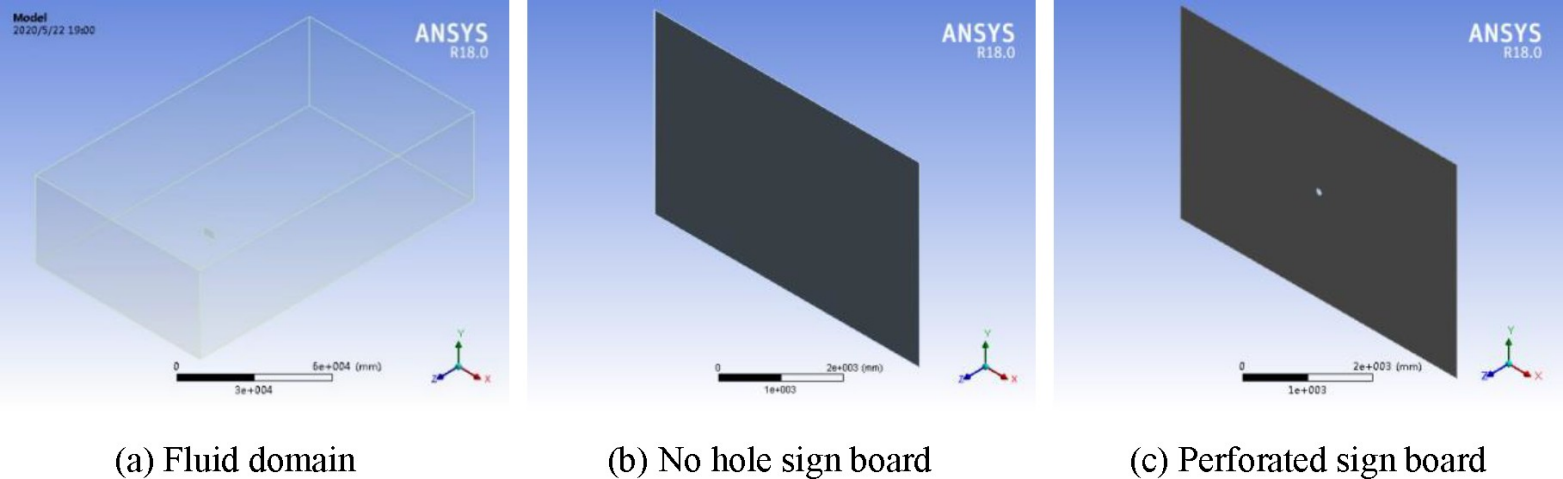
Fluid computing domain and sign board model.

For fluid and solid modelling, ANSYS DesignModeler constructs the fluid computational domain, the no hole sign board model, and the perforated sign board model. The calculation domain size of the flow field is 150 m long, 90 m wide and 42 m high. The fluid calculation domain and the sign board model are shown in Fig 1.

**Fig 2:**
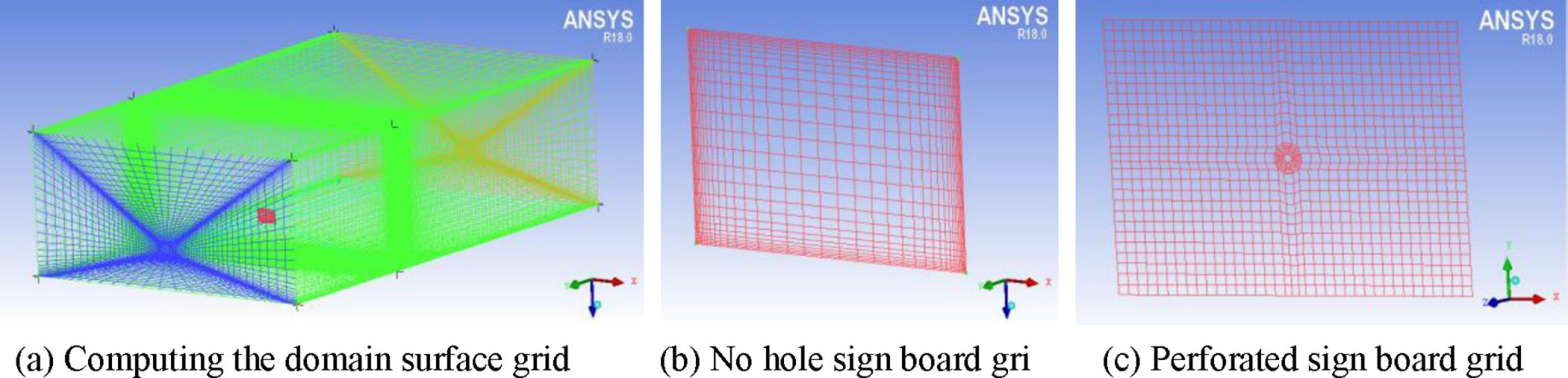
Structured grid generation results.

For grid division, the ICEM CFD module was used to divide the computational fields of the no-hole and open-hole sign boards. Grids are usually divided into structured grids and unstructured grids according to their data structure. The topological structure is equivalent to the uniform grid in a rectangular domain, which obtains a higher mesh quality and is suitable for simple geometric structures. Unstructured grids have no regular topology and no concept of layers, so they are flexible. However, unstructured grids require large amounts of memory for computation and are suitable for complex geometric structures. In the experiment, the sign board model was divided by structural grids, the target *y*^*+*^ value was set to 30, the height of the first-layer grid nodes was 0.0003 m, and the grid growth rate was 1.1. After grid division, the number of cells is 1.5 million, and the grid division results are shown in Fig 2.

Chao et al. [36] demonstrated the feasibility of CFD simulation by using the empirical formula for traditional sign board wind loads. The Reynolds average NS model (RANS) was selected to solve the turbulence in the wind-borne simulation process. Standard k-ε, RNG k-ε, and realisable k-ε models were selected and Fluent was used to simulate the wind load of the non-porous sign board at 22 m/s, 25 m/s, 30 m/s, 35 m/s, 40 m/s, and 45 m/s. The calculated field inlet is a velocity inlet, the outlet is free, and the reference pressure is 0 Pa. The four sides of the computational domain are sliding wall surfaces. The parameters for fluid turbulence intensity and turbulence length are shown in Table 2. The simulation results of different turbulence models were compared with the results obtained by empirical calculations, and the turbulence model with the minimum error was selected for subsequent wind load simulation with holes. The comparison of error results are shown in Tables 3–5. The results show that the average error of the RNG model under different wind speeds is 2.63%. Therefore, the turbulence model is suitable for this study.

**Table 2:** Fluent boundary condition settings.

Velocity (m/s)	intensity of turbulence (%)	Turbulence length dimension(m)
22	2.16311	2.55604
25	2.12882	2.55604
30	2.08085	2.55604
35	2.04114	2.55604
40	2.00735	2.55604
45	1.97801	2.55604

**Table 3:** Simulation error of standard k-ε.

Velocity (m/s)	Simulated pressure (N)	Calculated pressure (N)	error	Average error
22	12533.072	11982.136	4.598%	3.72%
25	16133.138	15472.800	4.268%	
30	22958.995	22280.832	3.044%	
35	31007.522	30326.688	2.245%	
40	41363.607	39610.368	4.426%	
45	52017.983	50131.872	3.762%	

**Table 4:** Simulation error of RNG k-ε.

Velocity (m/s)	Simulated pressure (N)	Calculated pressure (N)	error	Average error
22	12275.632	11982.136	2.449%	2.63%
25	15828.882	15472.800	2.301%	
30	23093.238	22280.832	3.646%	
35	31191.762	30326.688	2.853%	
40	40746.901	39610.368	2.869%	
45	50969.232	50131.872	1.670%	

**Table 5:** Simulation error of realisable k-ε.

Velocity (m/s)	Simulated pressure (N)	Calculated pressure (N)	error	Average error
22	12581.985	11982.136	5.006%	4.41%
25	15624.911	15472.800	0.983%	
30	23525.250	22280.832	5.585%	
35	31816.346	30326.688	4.912%	
40	41640.560	39610.368	5.125%	
45	52563.600	50131.872	4.851%	

**Fig 3:**
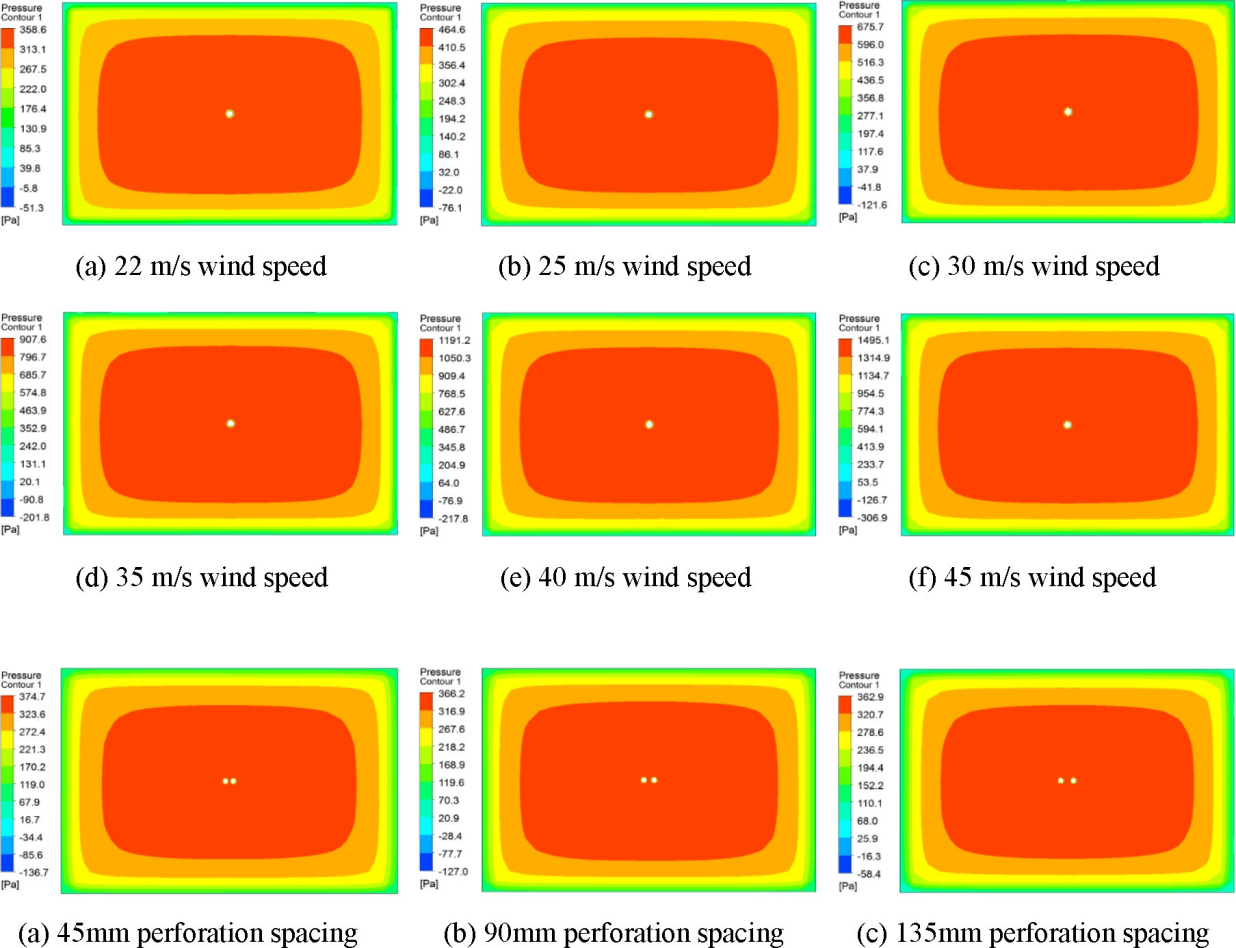
Wind pressure cloud chart of perforated traffic sign board with diameter of 120mm.

**Fig 4:**
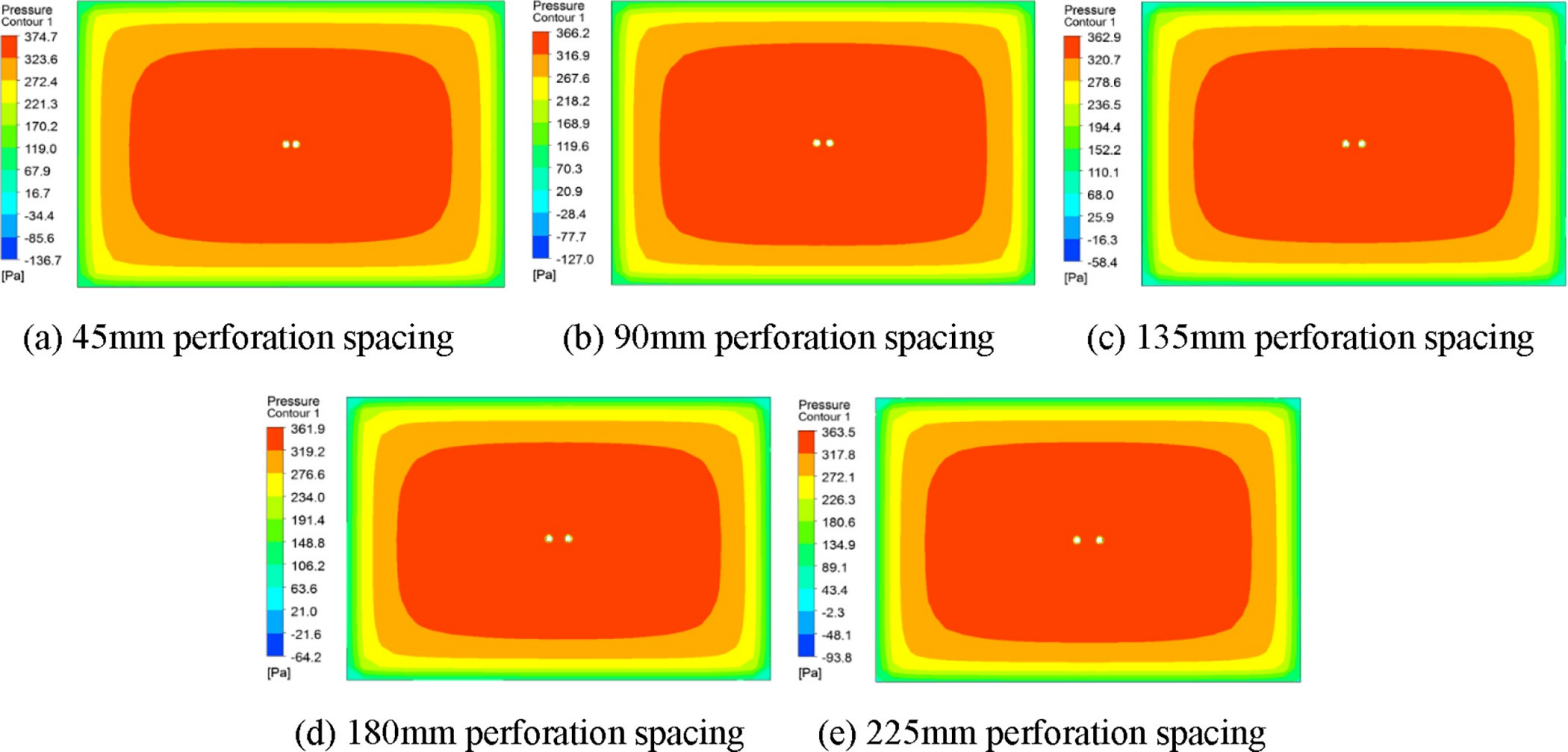
Wind pressure cloud charts of traffic sign plates with different perforation spacing.

Considering that the sign information font height is generally 60 mm and the spacing is 120 mm, the perforation diameter of sign board model is set to 30 mm, 60 mm, 90 mm, 120 mm, 150 mm, and 180 mm, and the hole spacing is set to 45 mm, 90 mm, 135 mm, 180 mm, and 225 mm. In Fluent, the RNG simulation model is used to simulate the wind load of sign boards with different perforation diameters and different hole spacings. Wind load simulations of 22 m/s, 25 m/s, 30 m/s, 35 m/s, 40 m/s, and 45 m/s wind speeds were performed on sign plates with different perforation diameters. For sign boards with different hole spacings, the perforation diameter is 90 mm and the wind speed is 22 m/s to simulate the wind load of the sign board. The hole spacing is set to five different values, which are 1 to 5 times the perforation radius. Fluent simulation results were imported into CFD-Post for result post-processing, and the wind pressure cloud map of the sign board surface was obtained. The wind pressure cloud map of a single perforated traffic sign plate, taking the perforated diameter of 120mm as an example, see Fig 3. The wind pressure cloud map of the sign boards with different hole spacings is shown in Fig 4.

## 3. Results analysis

### 3.1. Wind pressure characteristics of perforation diameter

#### 3.1.1. Influence of perforation diameter on the wind pressure distribution

**Fig 5:**
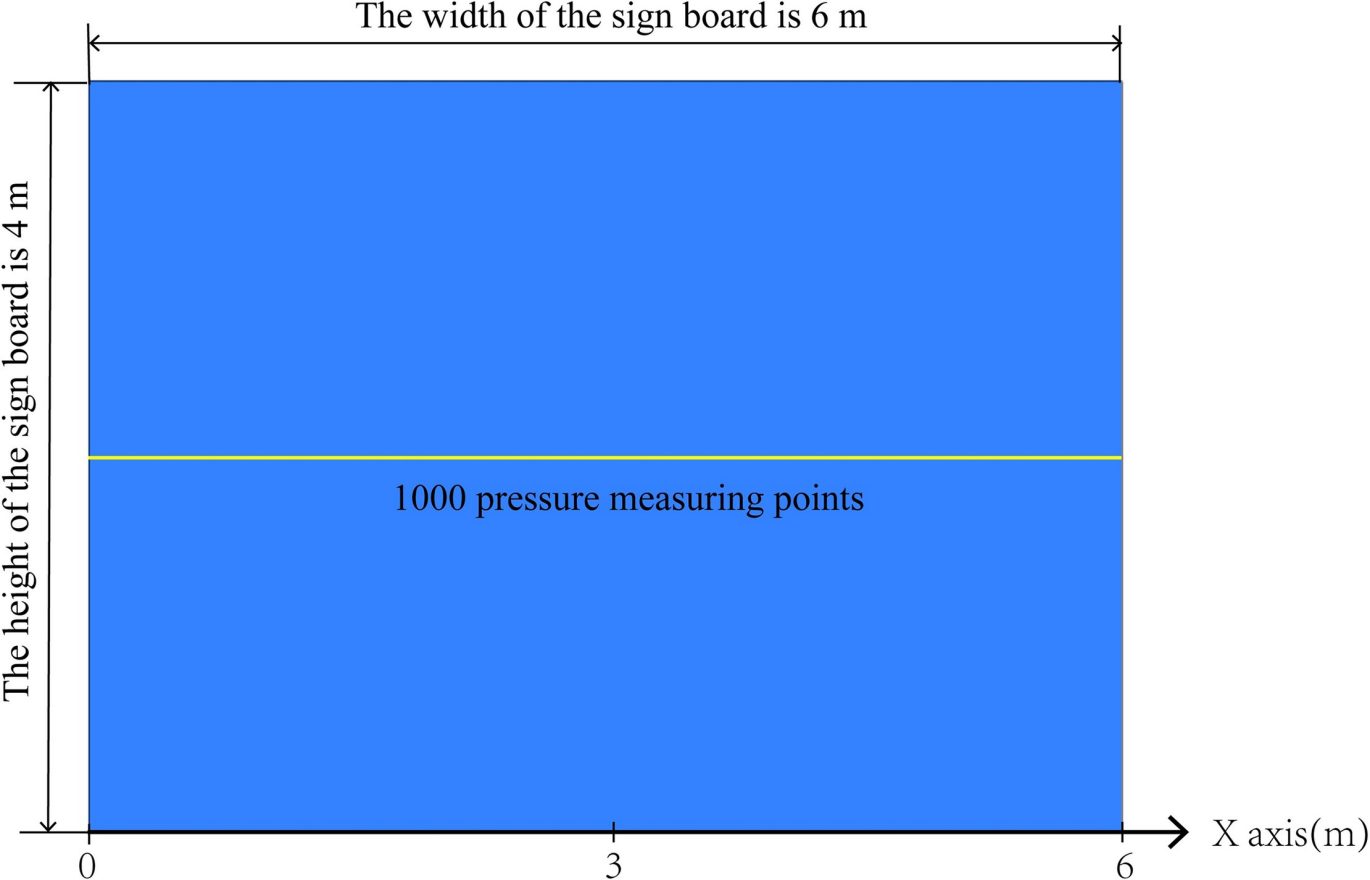
The arrangement of wind pressure measuring points on the surface of traffic sign board.

**Fig 6:**
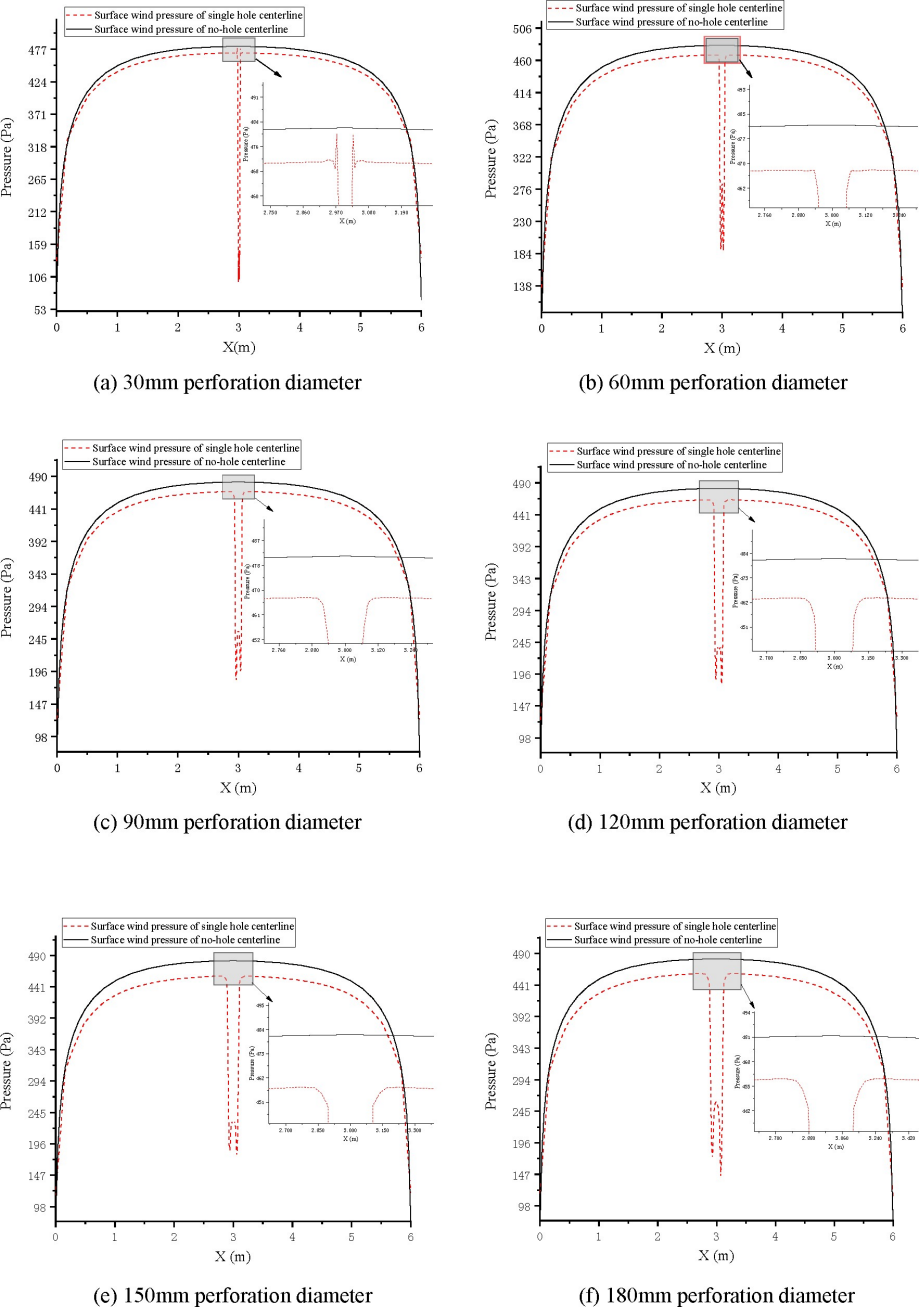
Comparison of wind pressure changes on centerline of perforated traffic sign board with 25 m/s wind speed.

With the *x*-axis representing the width of the marker, the *x*-axis coordinate corresponding to the centre of the hole is *x* = 3. In CD-POST, 1000 pressure measuring points (as shown in the yellow line in Fig 5) were arranged on the horizontal midline of the sign board to extract the surface wind pressure data. The broken line diagrams of extracted wind pressure data, taking 25m/s wind speed as an example, see Fig 6.

It can be seen from Fig 6 that, for the non-porous sign board, the closer to the centre, the higher the wind pressure. The overall wind pressure showed a trend of being high toward the centre and low toward the sides. Near the centre, the surface wind pressure gradually tends to be stable. The wind pressure on the overall surface of the perforated sign board is lower than that on the non-perforated sign board. The closer to the central region, the wind pressure difference between the two gradually increases. When the diameter of the hole is 30 mm, the wind pressure from the edge of the hole to the centre of the round hole temporarily surges before falling back quickly. With the increase in the perforation diameter, the surge of wind pressure from the edge of the hole to the centre of the round hole disappeared and the wind pressure change gradually slowed down near the edge of the hole. From the center of the hole circle to the edge of the hole, and the wind pressure is high in the middle and low on both sides, but the overall wind pressure is lower than at the periphery of the hole.

**Fig 7:**
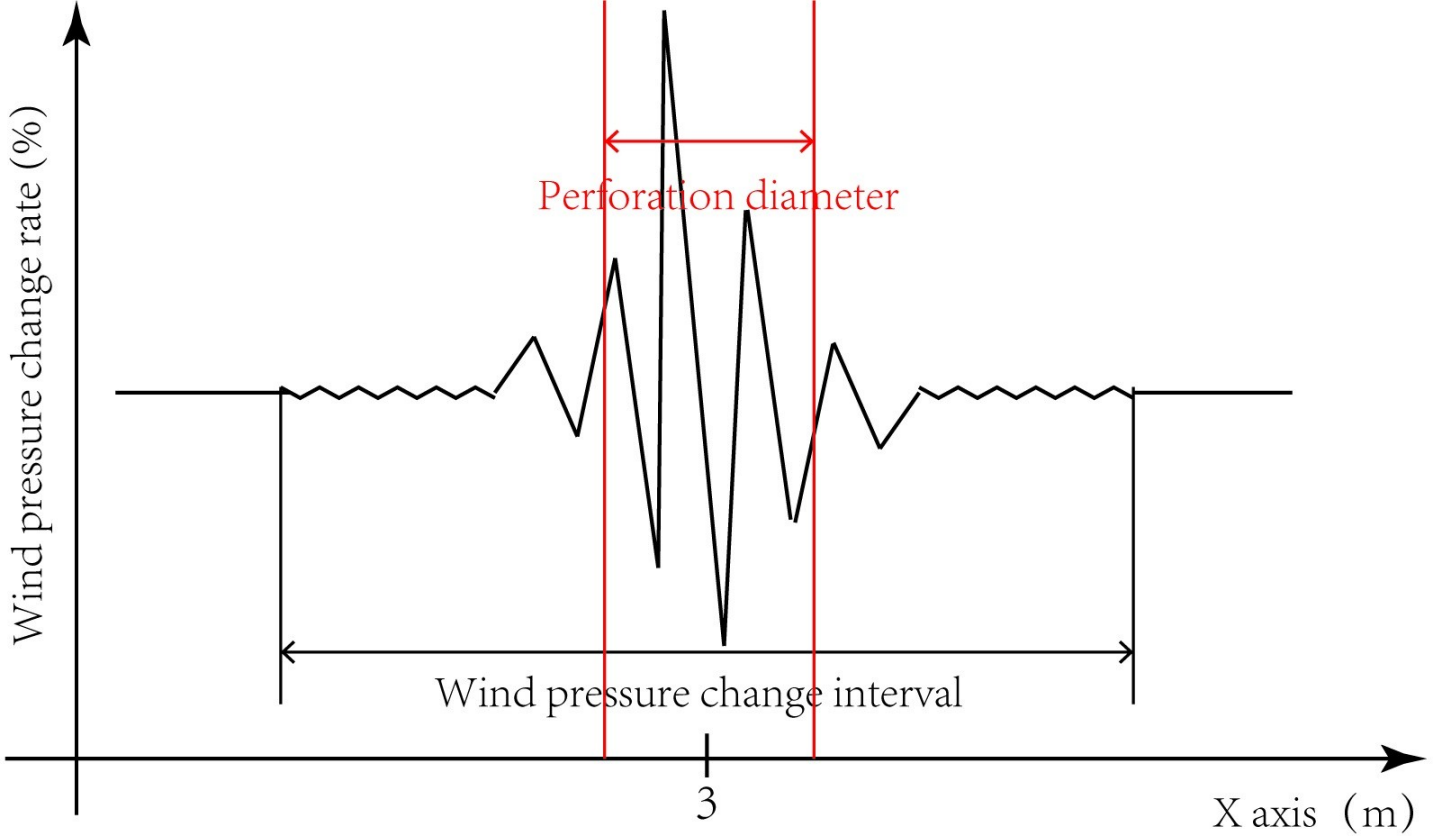
Wind pressure change interval of perforation diameter.

The wind pressure on the surface of the non-porous sign board tends to be stable in the central region. After perforation, the closer to the edge of the perforation, the more evidently the surface wind pressure change rate is affected. The wind pressure data obtained from 1000 measurement points were processed and revealed sequential growth in the direction of the *x*-axis increase. Because the x-axis coordinate corresponding to the centre of the perforated circle is x = 3 (unit is m), values closer to x = 3 correspond to larger variation in wind pressure amplitude. On both sides of x = 3, there is an initial change point of the wind pressure change rate, corresponding to the x-axis coordinate values of x_1_ and x_2_. The value of |x_1_ − x_2_| is defined as the wind pressure change interval of the perforation diameter, as shown in Fig 7. The wind pressure change interval for different perforation diameters is shown in Table 6.

**Table 6:** The initial change interval of wind pressure for different perforation diameters.

Perforation diameter (mm)	Wind pressure change interval (mm)		
	X_1_	X_2_	|X_1_-X_2_|
30	2931	3075	144
60	2859	3153	294
90	2829	3183	354
120	2781	3225	444
150	2715	3267	552
180	2691	3315	624

**Fig 8:**
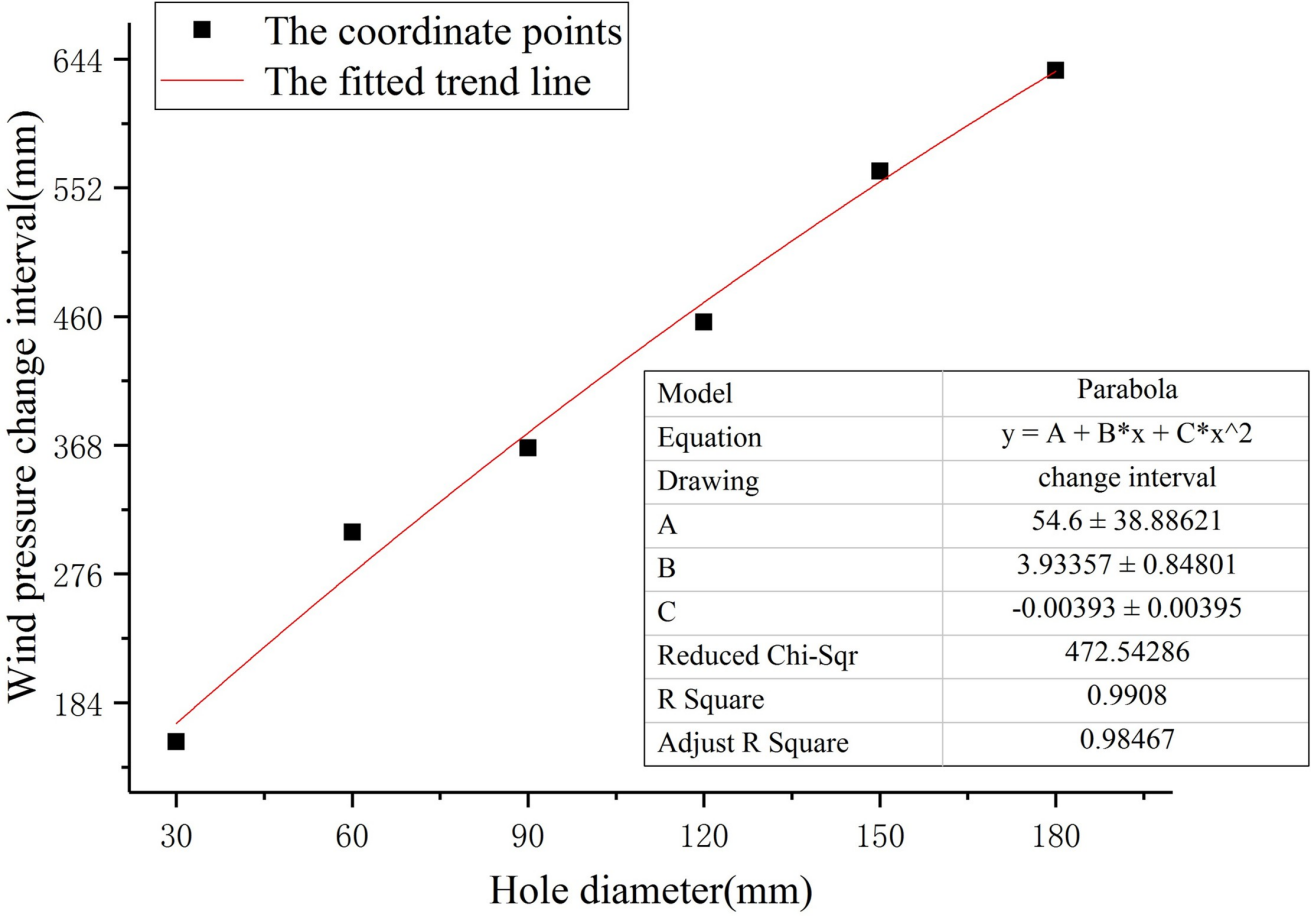
Fitting of wind pressure change interval trend.

**Fig 9:**
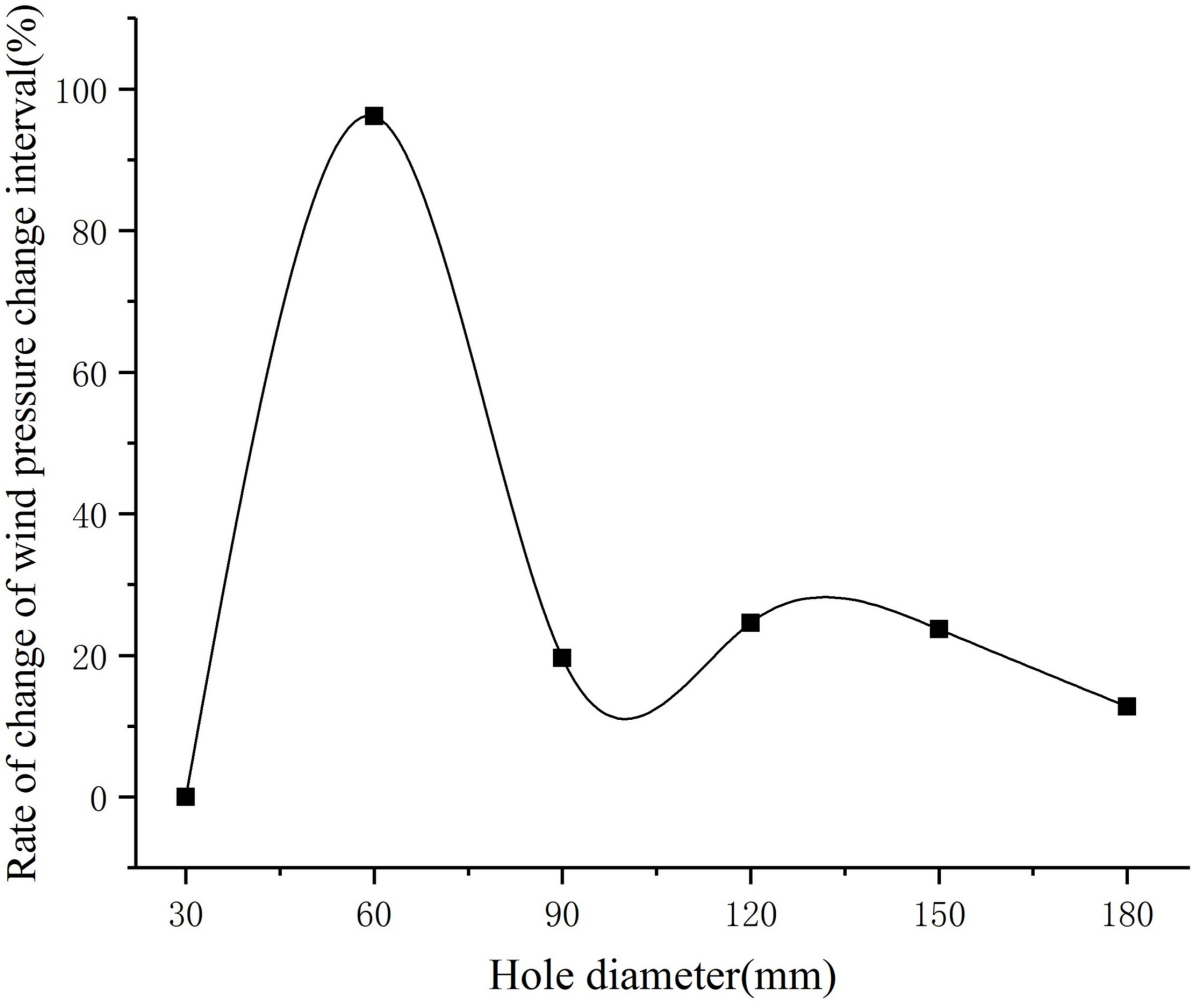
Rate of change of wind pressure change interval.

**Fig 10:**
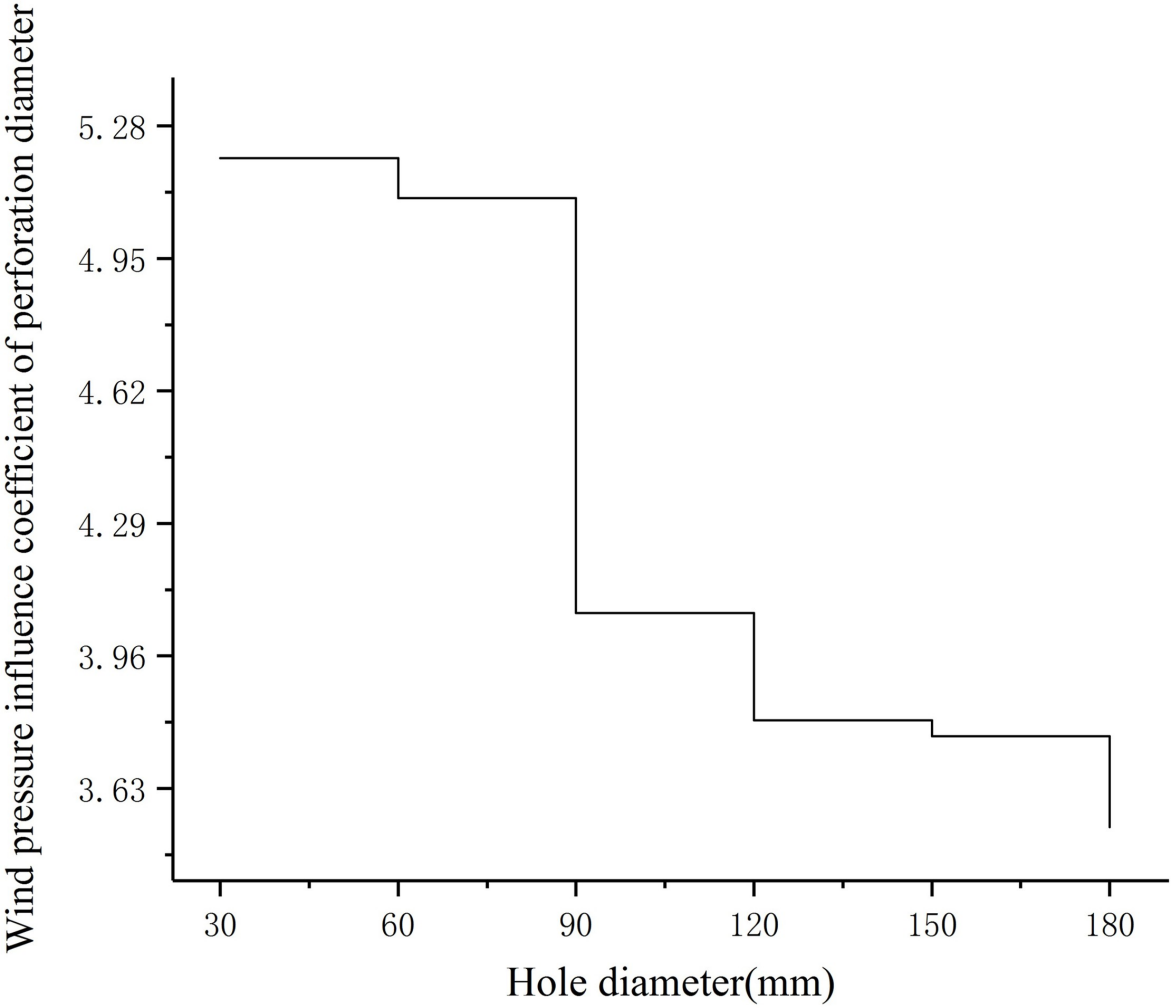
The wind pressure influence coefficient of perforation diameter.

Coordinate points and fitting trend lines of different perforation diameters corresponding to the wind pressure change interval are shown in Fig 8. The growth curve of the wind pressure change interval corresponding to different perforation diameters is shown in Fig 9. The wind pressure change interval is divided by the perforation diameter, and the result is defined as the wind pressure influence coefficient of perforation diameter, which reflects the degree of influence of different perforation diameters on the wind pressure change interval. A larger wind pressure influence coefficient of perforation diameter corresponds to a more significant influence of the perforation diameter on the wind pressure change interval. The wind pressure influence coefficient of perforation diameter is shown in Fig 10.

It can be seen from Fig 8 that the perforation diameter and the wind pressure change interval have a quadratic function correlation. When the perforation diameter is small, the wind pressure change interval increases rapidly. With the increase in the perforation diameter, the wind pressure change interval tends to be flat. Fig 9 shows that the wind pressure change interval has the fastest growth rate for aperture diameters between 30 mm and 60 mm. The growth rate of the wind pressure change interval for perforation diameters between 60 mm and 90 mm decreases significantly. Between the perforation diameter 90mm and 120mm, the wind pressure change interval first decreases and then increases. The growth rate of the wind pressure change interval between 120mm and 180mm perforation diameters slowed down gradually. As can be seen from Fig 10, when the perforation diameter is small, the wind pressure change interval is 4 to 5 times that of the perforation diameter. With the increase of the perforation diameter, the wind pressure influence coefficient of perforation diameter decreases. When the diameter of the hole is 60mm to 120mm, the wind pressure influence coefficient of perforation diameter decreases the fastest. When the diameter of the hole is bigger than 120mm, the wind pressure influence coefficient of perforation diameter decreases slowly.

#### 3.1.2. Analysis of downwind load characteristics of single hole traffic sign board

The CFD simulation results of the wind loads on the traffic sign board before and after perforation were compared to obtain the wind load reduction data of sign boards under different perforation diameters and simulated wind speeds. Correlation analysis of the perforation diameter, wind speed, and wind load reduction was carried out. Through three dimensional fittings, it was found that the wind load reduction is a binary quadratic equation related to the perforation diameter and wind speed. The surface fitting equation is shown in Eq (7).

The binary quadratic equation of wind load reduction as a function of perforation diameter and wind speed is given by:

$$0.01x^{2}-0.2y^{2}+0.44xy-9.66x+4.3y+75.92=z$$ (7)

where *x* is the perforation diameter, *y* is the wind speed, and *z* is the wind load reduction.

**Fig 11:**
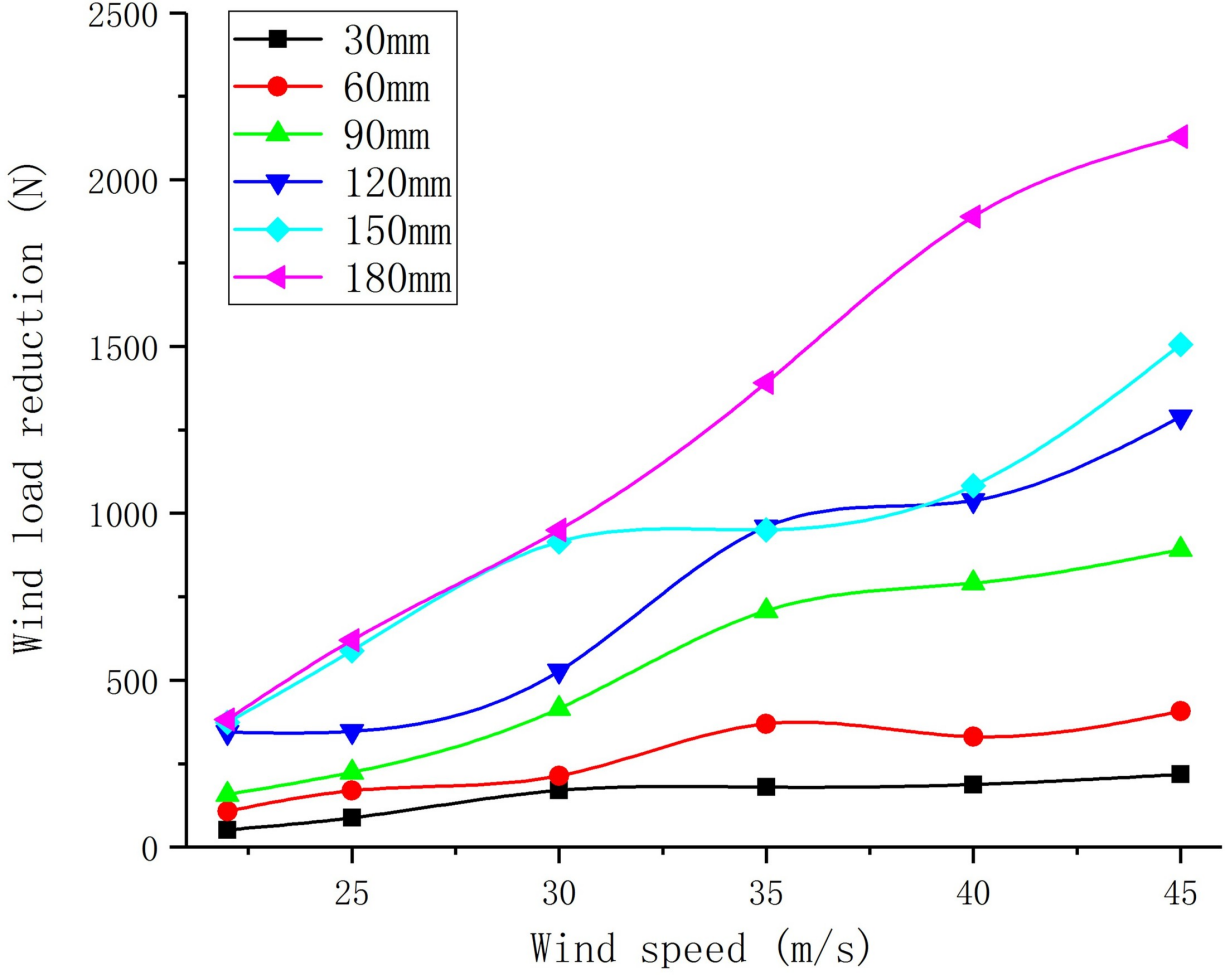
Wind load reduction curve of sign boards with different perforation diameters.

**Fig 12:**
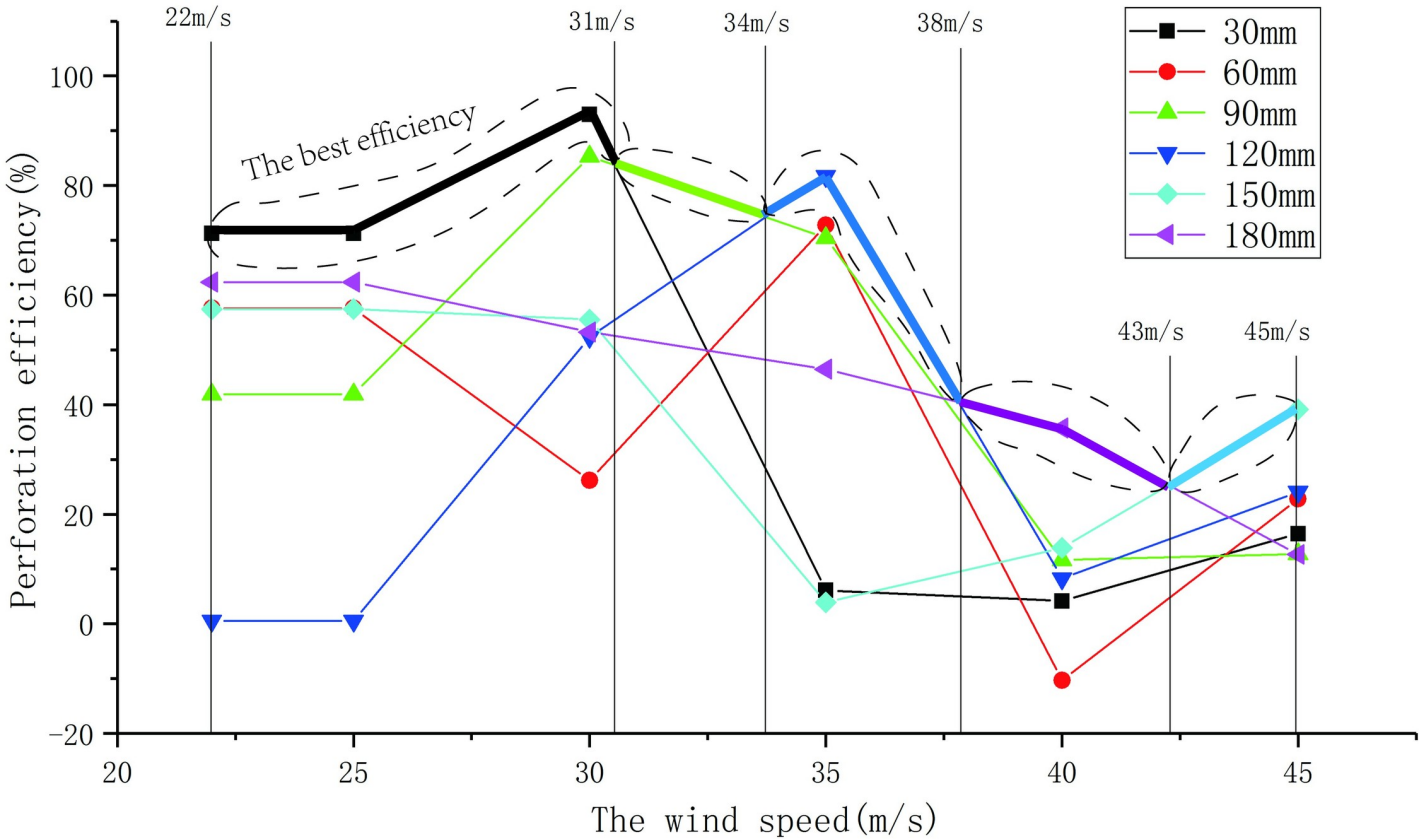
Perforation efficiency changes under different wind speeds.

The wind load reduction changes of the sign boards with different perforation diameters under different wind speeds are shown in Fig 11. Under different wind speeds, the rate of change of wind load reduction of the sign board with the same perforation diameter is defined as perforation efficiency. The variations in perforation efficiency with speed are shown in Fig 12. As shown in Fig 11, the variation of wind load reduction with different perforation diameters has the following patterns:

1. The reduction of wind load with different perforation diameters is proportional to the wind speed.
2. When the diameter of the perforation is between 30 mm and 60 mm, the wind load of the sign board has a small drop at different wind speeds.
3. When the perforation diameter is between 150 mm and 180 mm, the wind load reduction amplitude of the sign board is almost the same as when the wind speed is approximately 22 to 30 m/s. For wind speeds greater than 30 m/s, the difference between them decreases and then gradually increases.
4. When the wind speed is approximately 35 to 40 m/s, the wind load reduction difference between 120 mm and 150 mm perforation diameter is small.

According to Fig 12, the optimal perforation efficiency under different wind speed ranges can be found, as described below; suggestions for perforation under different wind speeds are shown in Table 7.

1. When the wind speed is approximately 22 to 31 m/s, the perforation efficiency with a perforation diameter of 30 mm is the highest.
2. When the wind speed is approximately 31 to 34 m/s, the perforation efficiency with a perforation diameter of 90 mm is the highest.
3. When the wind speed is approximately 34 to 38 m/s, the perforation efficiency with a perforation diameter of 120 mm is the highest.
4. When the wind speed is approximately 38 to 43 m/s, the perforation efficiency with a perforation diameter of 180 mm is the highest.
5. When the wind speed is approximately 43 to 45 m/s, the perforation efficiency with a perforation diameter of 150 mm is the highest.

**Table 7:** Recommended value of the best perforation diameter.

Wind speed range (m/s)	22–31	31–34	34–38	38–43	43–45
Optimum perforation diameter (mm)	30	90	120	180	150

According to the Beaufort Wind Scale, wind speeds of 35 m/s are equivalent to a category 12 typhoon. At a wind speed of 35 m/s, the sign plate perforation measures are ineffective, and this wind speed can cause significant damage to the support structure of the traffic sign plate. Considering the influencing factors such as hole diameter, wind speed, and visual recognition effect, some suggestions for the perforation measures are as follows:

1. In the areas near the sign information, the wind speed range is 22–31 m/s, and the hole diameter is 30 mm. If more significant wind load reduction is required, it can be widened to 60mm.
2. In the areas between the sign information and the edge of the sign plate, the wind speed range is 31–38 m/s, and the hole diameter is from 90 mm to 120 mm.
3. In the edge areas of the sign plate, the wind speed range is 38–45 m/s, and the hole diameter is from 180 mm to 150 mm.

### 3.2. Influence of perforation spacing on wind pressure distribution on the surface of traffic signs

**Fig 13:**
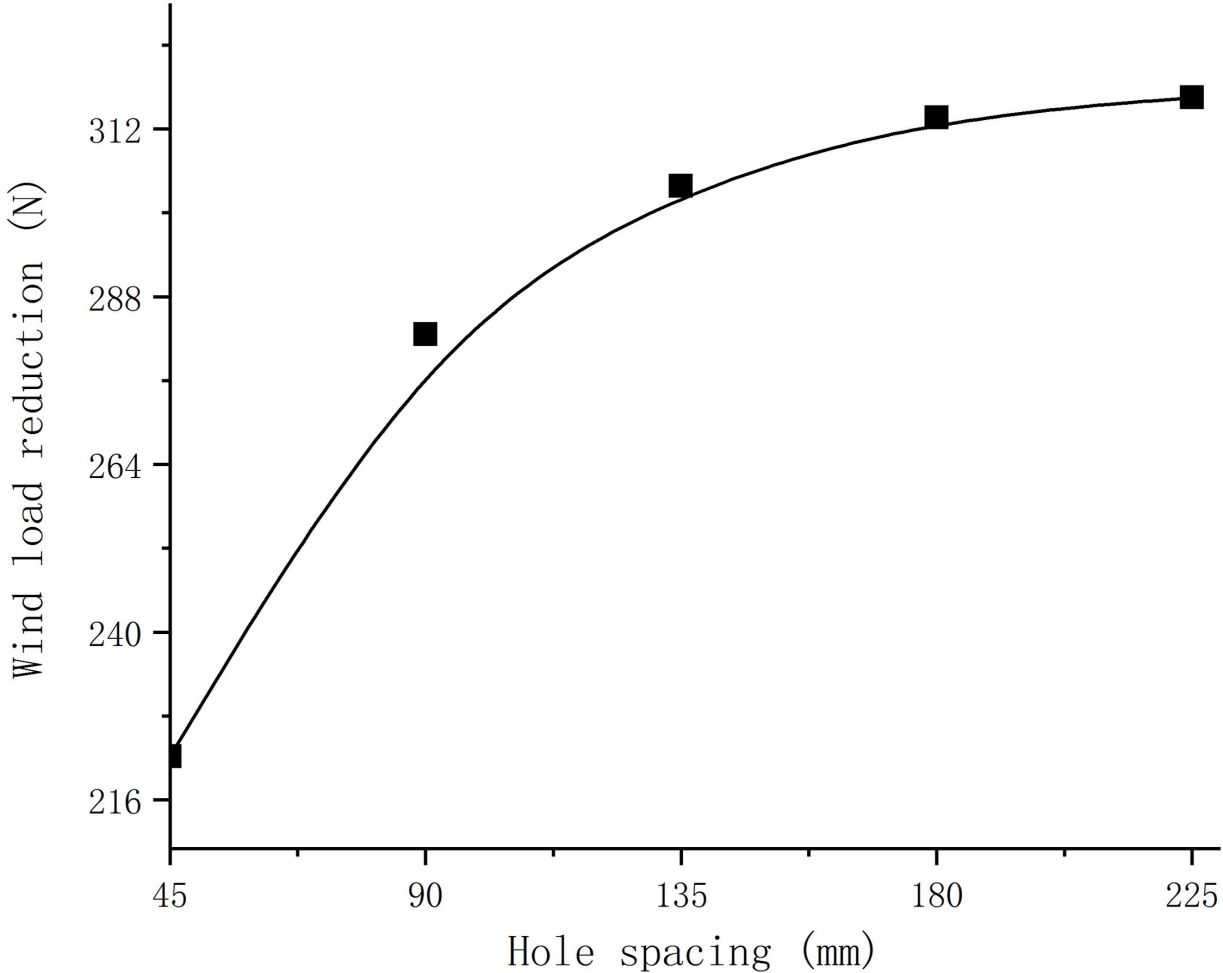
The relationship between perforated spacing of traffic sign board and wind load reduction.

**Fig 14:**
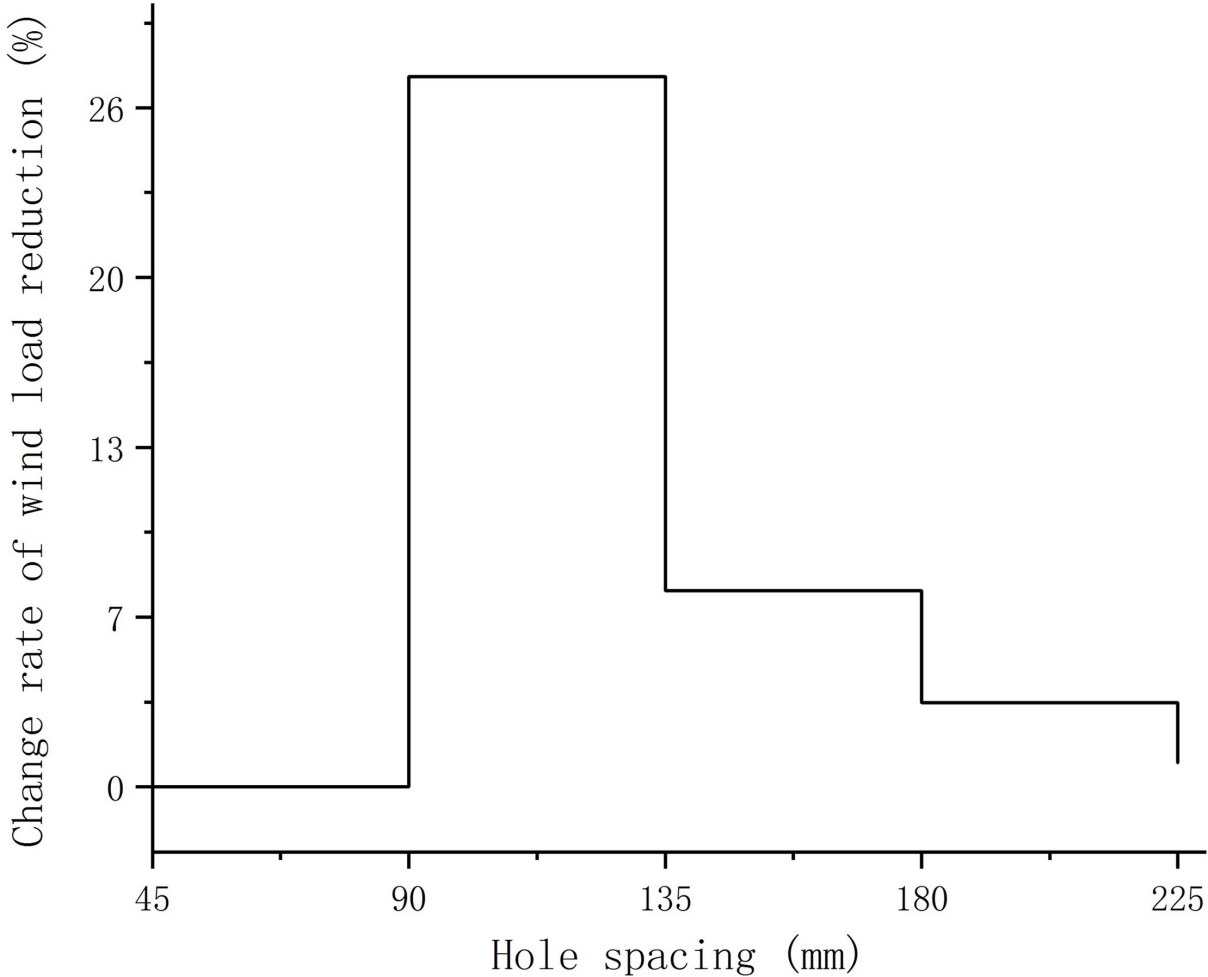
The relationship between perforated spacing of traffic sign board and the change rate of wind load reduction.

The CFD simulation results of sign boards with different hole spacings and non-hole spacings were compared to obtain the wind load reduction data for sign boards with different hole spacings. Fig 13 shows the relationship between the hole spacing and wind load drop and Fig 14 shows the relationship between the hole spacing and rate of change of wind load drop.

It can be seen from Figs 13 and 14 that when the hole spacing is from 45 mm to 90 mm, the wind load decline increases continuously and the growth rate reaches a peak. When the hole spacing is greater than 90 mm, the wind load decline continues to increase, but the wind load decline growth rate decreases rapidly. When the hole spacing is 135 mm to 180 mm, the wind-borne decline rate is dropping with a small change in the rate. When the hole spacing is 180 mm to 225 mm, the wind-borne decline rate further decreases and when the hole spacing is 225 mm the wind-borne decline rate approaches zero. To summarise, in the case of the same number of holes, when the hole spacing is small, the reduction in the wind load of the sign board is less than that of a board with a large hole spacing.

The perforation spacing affects the total number of perforations on the whole board. Considering the factors affecting the visual recognition and hole spacing of the marking plate, the suggestions for perforation are as follows:

1. For the areas near the sign information, the perforation spacing should be greater than 4–5 times the aperture radius.
2. For the areas between the sign information and the edge of the board, the hole spacing should be greater than 3 to 4 times the aperture radius.
3. For the edge of the board, the spacing of perforation should be greater than 3 times the radius of perforation.

## 4. Conclusion and discussion

In this paper, ANSYS was used for CFD simulation of traffic sign boards to study the influence of perforation diameter and hole spacing on wind pressure distribution on the sign board surface under different conditions. First, the object of the study is abstracted and simplified physically. Secondly, wind-borne simulation experiments for non-perforated sign boards, wind-borne simulation experiments for sign boards with different perforation diameters, and wind-borne simulation experiments for sign boards with different hole spacings were designed. The ANSYS fluid analysis module was used for physical modelling, meshing, simulation solution, and result post-processing. Finally, the wind load simulation results of the three sign board models were extracted for comparative analysis, and the influence of different perforation diameters and hole spacing on the wind load decline of the sign board was studied.

Through the analysis of experimental results and wind pressure data, the following conclusions are established:

1. The wind pressure distribution on the non-perforated sign board shows high wind loading in the centre and low in the edge.
2. The wind pressure distribution is high in the middle and low on both sides towards the edge of the hole circle, but the overall wind pressure is lower than that around the hole.
3. When the diameter of the perforation is small, the outward wind pressure at the edge of the perforation experiences a temporary surge, which disappears with the increase of the aperture diameter.
4. The perforation diameter and the wind pressure change interval have a quadratic function correlation.
5. When the diameter of the perforation is between 30 and 60 mm, and the wind pressure change interval grows the fastest. The growth rate of the wind pressure change interval for perforation diameters between 60 mm and 90 mm decreases significantly. Between the perforation diameters of 90 mm and 120 mm, the wind pressure change interval first decreases and then increases. The growth rate of the wind pressure change interval between 120mm and 180mm perforation diameters slowed down gradually.
6. The reduction of wind load with different perforation diameters is proportional to the wind speed. When the diameter of the perforation is between 30 mm and 60 mm, the wind load of the sign board has a small drop at different wind speeds. When the perforation diameter is between 150 mm and 180 mm, the wind load reduction amplitude of the sign board is almost the same as when the wind speed is approximately 22 to 30 m/s. For wind speeds greater than 30 m/s, the difference between them decreases and then gradually increases. When the wind speed is approximately 35 to 40 m/s, the wind load reduction difference between 120 mm and 150 mm perforation diameter is small.
7. The relationship between perforation diameter, wind speed, and wind load reduction satisfies a binary quadratic equation.
8. In the case of the same number of holes, when the hole spacing is small, the reduction of wind load of the sign board is less than that of the hole plan with a large hole spacing.

Combined with the characteristics of wind pressure distribution, the following suggestions are provided for the perforation scheme of traffic sign plates:

1. Close to the sign information area, the wind speed range is 22–31 m/s, then the diameter of the hole is recommended to be 30mm. If greater wind load reduction is required, it can be widened to 60mm. In the areas between the sign information and the edge of the board, the wind speed range is 31–38 m/s, then 90mm to 120 mm perforation diameters are recommended. For the edge area of the signboards, the wind speed range is 38–45 m/s, then a perforation diameter from 180mm to 150 mm is recommended.
2. Close to the sign information area, it is recommended that the hole spacing be greater than or equal to 4 to 5 times the radius of the hole. In the area between the marking information and the edge of the board, a hole spacing 3 to 4 times the hole radius is recommended. On the edge of the sign board, hole spacings 3 times the hole radius is recommended.

The conclusions of this paper can guide practical engineering application and the theoretical analysis of wind load of sign boards. However, there are still some factors not considered in the experiment:

1. Fatigue analysis was not performed to verify whether the perforation scheme meets the structural strength requirements.
2. There was no study or evaluation of the visual identity of the perforated sign plates.

In subsequent studies, a one-way fluid-structure coupling analysis can be performed on the perforated sign board to study the deformation of the sign board under different wind speeds. In addition, the visual recognition evaluation model of the perforated sign board can be established to optimise the perforated scheme.

## Supporting information

S1 File (ZIP)
